# On the Mechanism of Gene Amplification Induced under Stress in Escherichia coli

**DOI:** 10.1371/journal.pgen.0020048

**Published:** 2006-04-07

**Authors:** Andrew Slack, P. C Thornton, Daniel B Magner, Susan M Rosenberg, P. J Hastings

**Affiliations:** 1 Department of Molecular and Human Genetics, Baylor College of Medicine, Houston, Texas, United States of America; 2 Interdepartmental Program in Cell and Molecular Biology, Baylor College of Medicine, Houston, Texas, United States of America; 3 Department of Biochemistry and Molecular Biology, Baylor College of Medicine, Houston, Texas, United States of America; 4 Department of Molecular Virology and Microbiology, Baylor College of Medicine, Houston, Texas, United States of America; INSERM U571, France

## Abstract

Gene amplification is a collection of processes whereby a DNA segment is reiterated to multiple copies per genome. It is important in carcinogenesis and resistance to chemotherapeutic agents, and can underlie adaptive evolution via increased expression of an amplified gene, evolution of new gene functions, and genome evolution. Though first described in the model organism Escherichia coli in the early 1960s, only scant information on the mechanism(s) of amplification in this system has been obtained, and many models for mechanism(s) were possible. More recently, some gene amplifications in E. coli were shown to be stress-inducible and to confer a selective advantage to cells under stress (adaptive amplifications), potentially accelerating evolution specifically when cells are poorly adapted to their environment. We focus on stress-induced amplification in E. coli and report several findings that indicate a novel molecular mechanism, and we suggest that most amplifications might be stress-induced, not spontaneous. First, as often hypothesized, but not shown previously, certain proteins used for DNA double-strand-break repair and homologous recombination are required for amplification. Second, in contrast with previous models in which homologous recombination between repeated sequences caused duplications that lead to amplification, the amplified DNAs are present in situ as tandem, direct repeats of 7–32 kilobases bordered by only 4 to 15 base pairs of G-rich homology, indicating an initial non-homologous recombination event. Sequences at the rearrangement junctions suggest nonhomologous recombination mechanisms that occur via template switching during DNA replication, but unlike previously described template switching events, these must occur over long distances. Third, we provide evidence that 3′-single-strand DNA ends are intermediates in the process, supporting a template-switching mechanism. Fourth, we provide evidence that lagging-strand templates are involved. Finally, we propose a novel, long-distance template-switching model for the mechanism of adaptive amplification that suggests how stress induces the amplifications. We outline its possible applicability to amplification in humans and other organisms and circumstances.

## Synopsis

A common change in genomes of all organisms is the reiteration of segments of DNA to multiple copies. DNA amplification can allow rapid evolution by changing the amounts of proteins made, and is instrumental in cancer formation, variation between human genomes, and antibiotic resistance and pathogenicity in microbes. Yet little is known about how amplification occurs, even in simple organisms. DNA amplification can occur in response to stress. In Escherichia coli bacteria, starvation stress provokes amplifications that can allow E. coli ultimately to adjust to the starvation condition. This study elucidates several aspects of the mechanism underlying these stress-provoked amplifications. The data suggest a new model in which DNA replication stalls during starvation, and the end of the new DNA jumps to another stalled replication fork to create a duplicated DNA segment. The duplication can then amplify to many copies by genetic recombination. This model, if correct, can explain how stress provokes these genome rearrangements—by replication stalling. The general model may be useful for other long-distance genome rearrangements in many organisms. Stress can cause rapid and profound changes in the genome, some of which can give cells an advantage—this paper helps to explain how.

## Introduction

Gene amplification is the reiteration of a segment of a genome. It is a manifestation of genomic instability that is found in many tumors, notably some cases of neuroblastoma and some breast cancer in which it is associated with poor prognosis [1,2], and that arises during tumor progression in many others [1,2]. Amplification (and reduction) of genomic segments is now also appreciated to be among the most common of sequence variations, both pathogenic and polymorphic, between individual human genomes [3–5]. Amplification also occurs in microbes, in which it is implicated in the evolution of pathogenesis and antibiotic resistance [6]. In eukaryotic cells, at least some amplification appears to arise by a breakage–fusion–bridge cycle [7–12], or by formation by an over-replication mechanism of extrachromosomal replicons (double minutes) that then multiply or reintegrate ectopically [13,14]. There is also a mechanism that appears to reiterate a genomic segment in situ [15], as also seen in bacteria (see [16]). The molecular mechanism underlying each of these amplifications remains obscure [15].

Amplification was described in the model organism Escherichia coli more than forty years ago, as unstable genetic changes linked to the *lac* locus that caused overproduction of the *lac*-encoded beta-galactosidase [17,18]. Subsequently, amplified *lac* DNA was shown to occur as direct repeats and to manifest instability dependent on homologous recombination protein, RecA [16], as predicted by the idea that unequal recombination of the repeats produced instability [17,18]. Recombination was hypothesized to be part of the amplification mechanism itself, but whether or not it was had not been determined. The only other clue about the mechanism for these E. coli amplifications was that the directly oriented, amplified genomic sequences were bordered by regions of very short homology, indicating an initial “nonhomologous” recombination event [19,20]. Because the amplifications studied were selected as specific fusions of promoterless genes to functional promoters, and so could occur in limited DNA regions, it was not clear how general this structure might have been. Surprisingly, little else has been revealed since then about how amplification in E. coli might arise. These amplifications were thought to arise spontaneously and to be merely revealed by selection of colonies with gene activity, although the authors recognized that amplification might occur only or preferentially under selection for the amplification [16], i.e., adaptively. Later work on mammalian amplification revealed the clear existence of spontaneous amplifications in culture cells [21]. By contrast, methotrexate resistance mediated by amplification of the dihydrofolate reductase locus appears to be induced by the drug treatment [22,23].

More recently, tandem gene amplification in E. coli has been shown to occur adaptively, as a response to conditions in which increased activity of the *lac* gene is selected [24]. These amplifications are formed after selection for many gene copies has begun, during starvation of cells with a weakly-functional (promoter-containing) *lac* gene on lactose medium. These appear to be stress-induced amplifications—induced by the starvation-stress condition—and indeed, their formation requires the cell's major starvation- and general-stress response [25], although which component(s) of that response are required is not known. This stress inducibility may facilitate genetic change specifically when organisms are poorly adapted to their environment, potentially accelerating evolution. As one of very few experimental systems in any organism in which adaptive, stress-induced amplification has been demonstrated rigorously, this system provides an important model for many instances of (particularly tandem) gene amplifications that might also include stress-inducible–mechanism components. Adaptive, stress inducibility could be important in amplifications that promote resistance to antibiotics [26] and chemotherapy [27], both of which stress cells, and amplifications that lead to loss of growth control or promote tumor progression [1], both of which can be viewed as selected events at the cellular level [28]. Moreover, human genomes are replete with structural variations between individuals [3,4,29], and these variations might have arisen by a stress-inducible mechanism. On an evolutionary scale, chromosomal structural differences between primates have been associated with microhomology-mediated events, similar to those described below [30], possibly implying an origin similar to that causing amplification in this system. Comparison of plant genomes between rice species shows the same phenomenon [31].

Little is known about the mechanism of the stress-induced amplifications in E. coli. The amplifications occur in situ, not extrachromosomally or ectopically, and are tandem repeats of varying sizes [24]. Stress-induced amplification requires induction of component(s) of the general- and starvation-stress response of E. coli [25]; it also requires some activity of the E. coli DNA-repair and lagging-strand DNA polymerase, Pol I [32]. That is nearly all that was known that bears on the mechanism of formation.

In the same starving cultures that produce stress-induced *lac* amplifications, a different pathway occurs, leading to reversion of the leaky *lac* frameshift allele (called “point mutagenesis” to distinguish it from genome rearrangements like amplification). The point mutations also allow growth on lactose medium and are also stress-induced [25,33–35]. The mutations were thought initially to be derived from gene amplifications [36], although recent work supports models in which stress-induced amplification and point mutagenesis are independent outcomes and mechanisms [32,37] (see below). In either model, amplification occurs, and its mechanism of formation is poorly understood.

Here we report several features of the mechanism of stress-induced amplification in E. coli that suggest a novel mechanism for tandem amplification induced under stress and for how stress induces genome rearrangement. First, we show that certain homologous recombination proteins function in amplification. Specifically, those that carry out DNA double-strand break repair (DSBR) are required. Second, contrary to a previous model (see [38]), however, repeats are not formed by an initial homologous recombination event between dispersed repeated sequences. Rather, the novel junctions at the sites of the repeats result from nonhomologous recombination in direct repeats of G-rich microhomologies of only 4 to 15 base pairs (bp). These are similar to sequences implicated previously in template-switching reactions during DNA replication that generate other nonhomologous recombination events (reviewed in [39]). However, the microhomologies reported here are separated by 7 to 32 kilobases (kb), a distance much greater than previously observed in template-switching events. Third, we propose a novel, long-distance template-switching model for the mechanism of formation of the repeated DNA that leads to stress-induced amplification. Fourth we support this model with evidence that 3′-single-strand (SS) DNA ends are molecular intermediates in the process, and fifth, provide evidence that lagging-strand templates are involved. The long-distance template-switch model suggests a possible molecular basis of the stress inducibility of these and other genome rearrangements. Similar models could explain some human genome segmental amplifications and other rearrangements [5,15,29,30], and other cases of tandem amplification. Finally, the similarity of the repeat borders to those seen previously in E. coli amplifications thought to be spontaneous suggests that perhaps most amplifications in E. coli are stress-induced, provoking genome evolution preferentially during poor adaptation to the environment.

## Results/Discussion

### DSBR/Homologous Recombination Proteins in Stress-Induced Amplification

**Figure 1:**
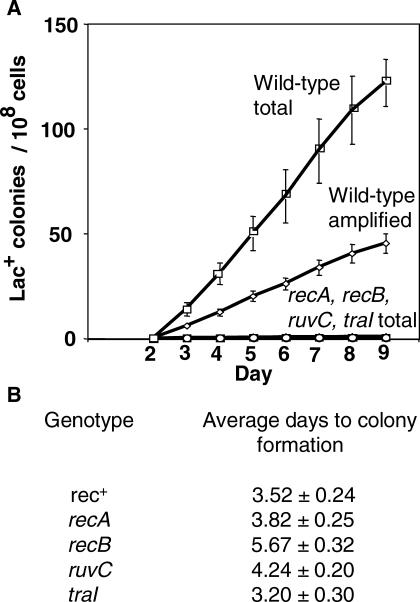
Homologous Recombination/DSBR Proteins Are Required for Adaptive Amplification These graphs, and similar ones below, show cumulative plots of Lac^+^ colonies arising per day in three or four parallel cultures under starvation on lactose minimal medium. The error bars show 1 SEM. See Table 2 for a list of all strains used. (A) *recA, recB, ruvC,* and *traI* mutants show severe depression of both point mutation and amplification under starvation. “Total” wild-type colonies (squares) are all Lac^+^ colonies, whether point mutant or *lac* amplified. *lac*-amplified colonies (diamonds) are distinguished from point mutants among the total colonies as previously described (see [16,24,32]) by picking total colonies from the lactose selection plates and streaking onto rich medium with X-gal dye on which *lac*-amplified clones form sectored blue and white colonies (see Figure 7), whereas point mutants form solid blue colonies. Strains used are as follows: “wild-type” *(rec^+^)* (squares and diamonds), SMR4562; *recA* (circles), SMR624; *recB* (triangles)*,* SMR593; *ruvC* (inverted triangles), SMR789; and *traI* (lowercase phi), SMR5232. Many of the symbols for mutant strains are obscured by others. (B) A reconstruction experiment. Mutant strains of the same five F′ factors carrying *lac* amplifications of various lengths were replated on lactose minimal medium with neighbor cells to determine growth rate [24], expressed as the average number of days (± SEM) taken to form visible colonies. This shows that the slow growth rate of mutant cells carrying amplification is not sufficient to explain the very low yield of amplified colonies seen in (A). Mutant *lac-*amplified strains are PJH428–PJH447 and PJH453–PJH457.

In the E. coli Lac assay [33], cells carrying a chromosomal *lac* deletion and a *lac* frameshift allele on an F′ conjugative plasmid are spread onto solid, minimal lactose medium. Lac^+^ colonies arise continuously over time during starvation. Those appearing during the first week are mostly compensatory frameshift revertants [40,41], whereas those appearing later consist of an increasingly larger fraction of stress-induced *lac*-amplified clones, with day-8 colonies represented by about 40% *lac*-amplification bearers (as observed previously [24,42]; see Figure 1).

Loss of components of the RecBC system of homologous recombinational DSBR, specifically, RecBC, RuvABC, and RecA, causes dramatic reduction in stress-induced total Lac^+^ colonies [43–45] (Figure 1). These include both stress-induced point mutants and *lac*-amplified clones. There is also a strong requirement for TraI, a single-strand endonuclease required for plasmid transfer [37] (Figure 1). TraI can be substituted for by a double-strand endonucleolytic cut delivered by the I-SceI restriction enzyme in vivo [37], implying that the role of TraI is in generating DNA breaks that become double-strand ends (DSEs), which promote point mutation, (and not by promoting transfer, which cannot occur with a linearized conjugative plasmid).

Whether these genetic requirements for DSBR proteins and TraI apply to the amplification mechanism cannot always be determined directly by assaying mutant strains for reduced numbers of late *lac*-amplified colonies, because some of the mutant strains are defective in deamplification [16], for example *recA* (below; Figure 1, legend). Deamplification gives rise to visible colony-color sectoring, which is how *lac* amplification is usually scored. This problem prevented others previously from determining whether homologous recombination proteins were required for *lac* amplifications thought to be spontaneous [16]. For stress-induced *lac* amplification, the magnitude of the reduction of total late Lac^+^ colonies in strains lacking RecA, RecB, RuvC, or TraI implies that both point mutation and amplification have been reduced or eliminated (Figure 1A). However, because even *rec^+^*–amplified isolates grow slowly and at variable rates on lactose medium [24], and *rec* and *ruv* mutants are slow growing, it was possible that the failure to see *lac*-amplified colonies in these mutant strains was caused by their slow growth rather than because amplification does not occur.

To distinguish these possibilities, we performed reconstruction experiments to determine how quickly or slowly *lac*-amplified strains lacking DSBR or TraI proteins form colonies. F′ factors carrying five different amplified arrays were transferred by conjugation into F^−^ strains carrying mutations in genes of the RecBC system. TraI cells carrying the same arrays were obtained by transducing Δ*traI* into the amplified isolates. *lac*-amplified cells of these genotypes were then plated under exact reconstructions of the conditions of an adaptive mutation experiment, and the time taken to form visible colonies was scored.

The greatest delay observed in colony formation for these genotypes relative to the Rec^+^ TraI^+^ parental strains was 2 d (Figure 1B). This is not sufficient to explain the observation of extremely low yield of *lac*-amplified colonies in these experiments (Figure 1A). Therefore, we can conclude that adaptive amplification, like adaptive point mutation, requires proteins of the RecBC DSBR pathway, including RecA, and the F-factor-encoded endonuclease, TraI.

### Direct Repeats/Microhomologies in Novel Junction Sequences

**Figure 2:**
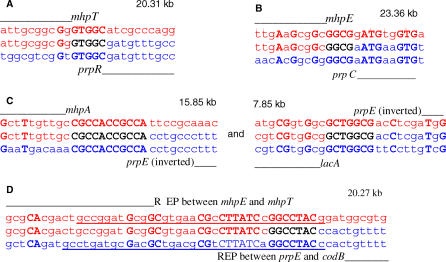
Examples of Novel Junction Sequences in Their Context See Table S1 for similar context information for all 31 junctions sequenced. One parental sequence with its gene designation is in red on the top line, the other in blue on the bottom line. The novel sequence is in the middle, with the junction sequence shown in black. Bold uppercase letters indicate identity between the parental sequences. The distances between the junction sequences on the parental chromosome are given in kb. REP repeated sequence elements are underlined. Strains used are as follows: (A) PJH4; (B) PJH23 and PJH37; (C) PJH39, the only complex amplicon, containing a direct and an inverted repeat, and hence two junctions; and (D) PJH32, an isolate with a REP repeated sequence element at the junction.

We identified the novel junction sequences between repeat units of 31 different stress-induced amplified isolates (Table 1). The positions of representative examples of the junctions with the lengths of their repeat units (amplicons) and some surrounding sequence are shown in Figure 2, and this information for all 31 junctions is given in Table S1.

**Table 1:**
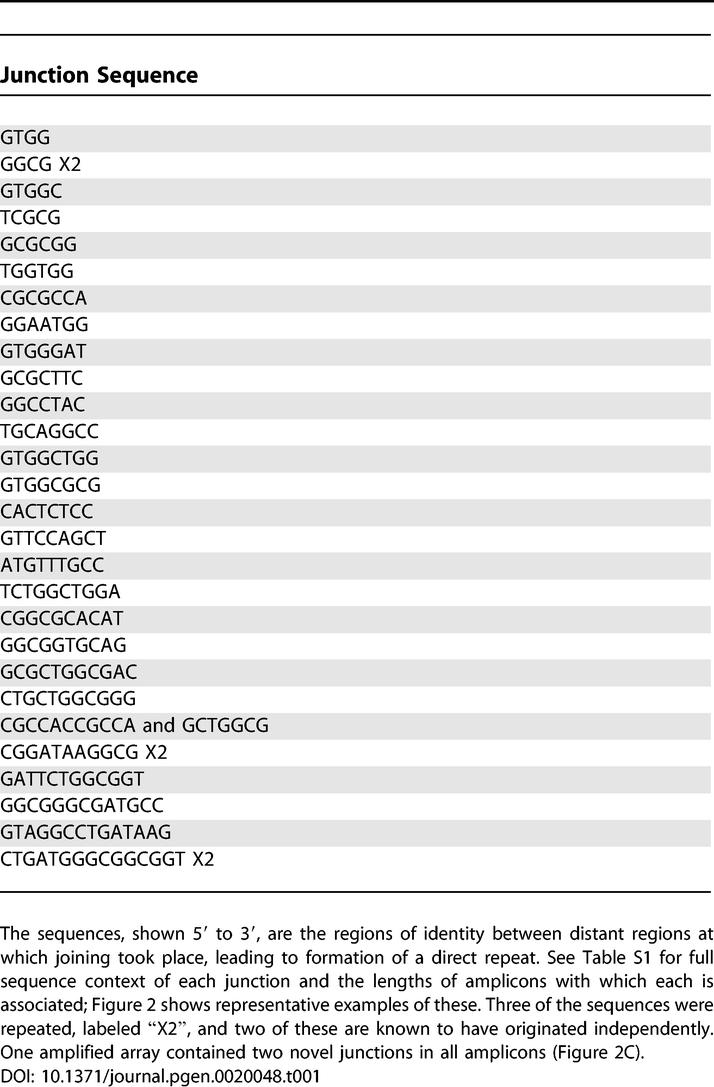
Sequences of 31 Novel Junctions from Stress-Induced Amplifications

**Table 2:**
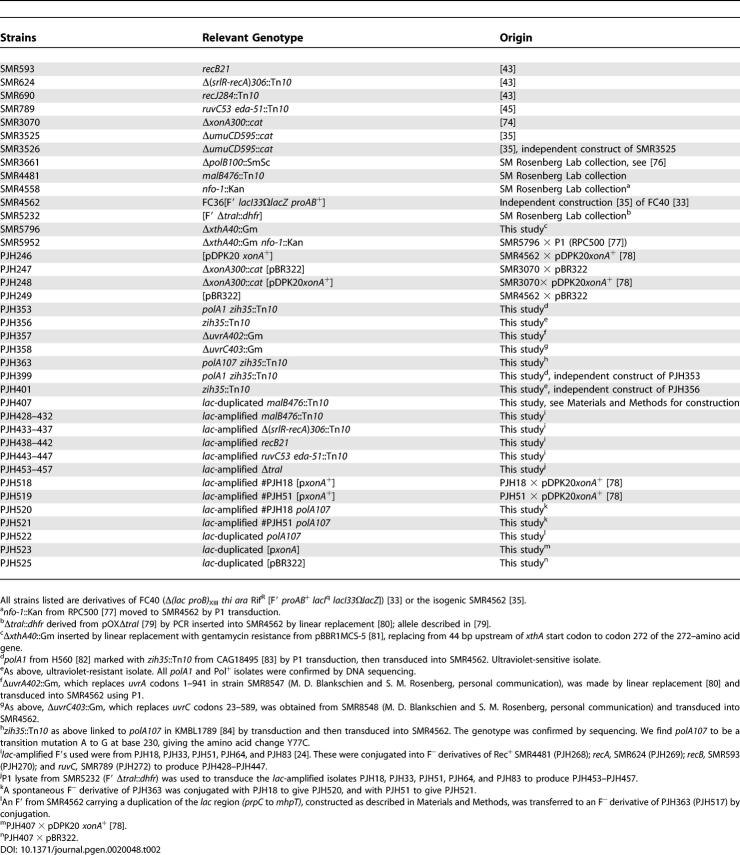
Origins of E. coli Strains Used

First, with one exception, there was a only a single, unique novel junction present, and all amplifications were oriented as direct repeats. One isolate has two novel junctions per amplification, with each amplicon having a direct repeat of 16 kb that includes the *lac* region, and an inverted repeat of about 8 kb that overlaps the 16 kb length (Figure 2C).

Second, many sites are used for these rearrangements, and the sequences of the junctions revealed a 4- to 15-bp length of sequence identity (Table 1; Figure 2, letters in bold). Some, but not all, of the junctions had further short identical regions separated from the junction identity by mismatches (“microhomologies”; Figure 2, uppercase letters; Table S1). There are 28 different junctions, three of which were isolated twice. In two of these three cases, the repeats came from different cultures and are therefore known to be independent. The junction sequence of four nucleotides that was isolated twice (Table 1, Figure 2B) has two further three-nucleotide lengths of identity separated by 1- or 2-bp mismatches. Microhomology junctions occurring more than once that were reported before for possible spontaneous amplifications [19] were at least ten nucleotides long if mismatches are allowed. Such a sequence is not expected to be repeated within 32 kb. On the other hand, we can see that such long sequence identity is not needed at other junctions; one of the junction sequences of only five nucleotides has no other identity nearby (Figure 2A). The E. coli genome contains repeated sequences, which were proposed to be sites of duplication via homologous recombination, leading to amplification [38]. However, the only repeat family represented, in only four of the junction sequences, is the REP sequence. REPs are non-identical 38-bp imperfect palindromes [46]. We found that the REP sequences involved are from different families [46], and that the length of perfect homology used in these recombination events was less than the length of the REP sequences. Thus, neither homologous recombination nor repeated elements is the most common precursor of duplications.

Two isolates showed point mutations adjacent to and to the left of the junction sequence. In one (PJH69, Table S1), the junction was associated with a 3-bp deletion, whereas the other (PJH83, Table S1) showed a 1-bp (cytosine) insertion. The meaning of these observed point mutations associated with rearrangement is not known.

Whereas the small imperfect homologies at the junctions are characteristic of rearrangements thought to occur by template switching during DNA replication, the distance between the microhomologies is far greater than the usual few hundred to few thousand bp of such rearrangements [39], which are thought to occur within a single replication fork. This leads us to suggest a novel kind of template-switching mechanism.

### DNA Intermediates: 3′-SS Ends Implicated

**Figure 3:**
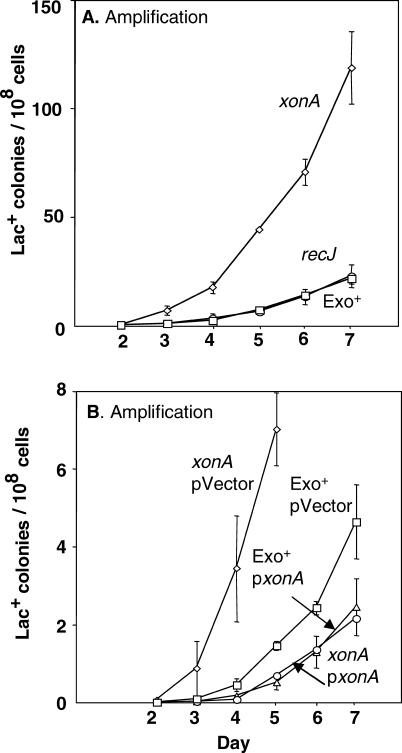
3′-SS DNA Ends Promote Stress-Induced Amplification (A) Deletion of *xonA,* encoding exonuclease I, causes a 2- to 6-fold increase in amplification. Disruption of *recJ,* encoding a 5′ exonuclease has no effect. Thus, increasing cellular levels of 3′-SS DNA ends increases stress-induced amplification. The associated strains are as follows: Exo^+^ (squares), SMR4562; *xonA* (diamonds), SMR3070; and *recJ* (circles), SMR690. (B) A plasmid carrying a wild-type *xonA* gene reduces amplification 2-fold below the level seen with vector alone, and approximately 10-fold below that in the Δ*xonA* background. Therefore 3′-SS DNA ends are required for, and thus appear to be intermediates in, the stress-induced amplification mechanism. The strains associated with each mutant are as follows: *xonA* pVector (diamonds), PJH247; *xonA* p*xonA* (circles), PJH248; Exo^+^ pVector (squares), PJH249; and Exo^+^ p*xonA* (triangles), PJH246. The overall reduction caused by ColE1-based plasmids generally is as observed previously [74].

Template switching is a property of 3′-SS DNA ends. The major 3′-SS–specific exonuclease in E. coli is ExoI, encoded by *xonA.* ExoI has been shown to influence deletion formation between short direct repeats in E. coli [47]. We find that deletion of *xonA* causes an increase in adaptive amplification of between 2- and 6-fold (Figure 3A). The mean of three experiments, plus or minus the standard error of the mean (SEM), is 4.0 ± 1.2. This implies that increasing cellular levels of 3′-SS ends increases stress-induced amplification. Mutation in *recJ,* which encodes the major 5′-SS–specific exonuclease, has no effect on amplification (Figure 3A). Moreover, in Figure 3B, we show that overproduction of ExoI leads to a 2-fold decline in the level of adaptive amplification (2.1-fold and 2.3-fold in two experiments), and an approximately 10-fold decline in the Δ*xonA* background. This supports the idea that decreasing cellular levels of 3′-SS ends below normal amounts decreases stress-induced amplification, and thus implies that 3-SS ends are a normal molecular intermediate in the stress-induced amplification process.

### Role of DNA Polymerase I, and Not Pols II, IV, or V: Lagging Strands Implicated

The novel junction sequences (Table 1, Figure 2) suggest a template-switching mechanism for formation of repeats in stress-induced amplifications, which is further supported by the evidence that 3′-SS ends are intermediates in stress-induced amplification (Figure 3). Because template switching might involve DNA polymerases other than the main replicative polymerase Pol III, we investigated the involvement of the remaining four E. coli DNA polymerases in stress-induced amplification. We found previously that the error-prone polymerase Pol IV, which is required for adaptive point mutation, is not required for amplification in this system [48]. We also reported a requirement for Pol I encoded by *polA* [32]. A *polA* temperature-sensitive mutant showed a 2- to 6-fold reduction in amplification at a semi-permissive temperature in repeated experiments. At the same time, adaptive point mutation was increased about 2-fold. We excluded the possibility that events were being channeled from the amplification pathway to the point-mutation pathway, via the known *polA*(Ts) phenotype of chronic induction of the SOS DNA-damage response [49], by showing that mutation of *dinB* (encoding Pol IV), the main cause of SOS-induced mutation in this system [48], does not affect the reduction in amplification seen in *polA*(Ts) strains [32]. Thus Pol I is required for adaptive amplification and not for point mutation, confirming that these are the endpoints of independent pathways.

**Figure 4:**
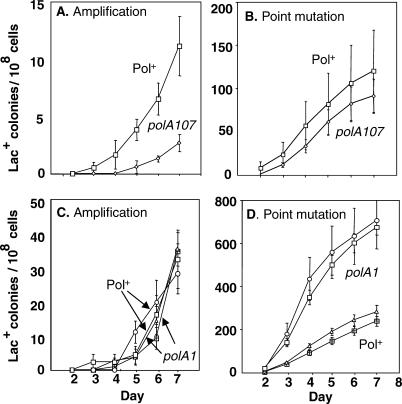
The Flap-Endonuclease Activity of DNA Polymerase I Promotes Adaptive Amplification with No Effect on Point Mutation: Lagging Strands Implicated (A and B**)** *polA107,* defective in the flap-endonuclease function of DNA polymerase I (Pol I), causes an approximately 5-fold depression of amplification and no effect on point mutation. Strains used are as follows: Pol^+^ (squares), PJH356; and *polA107* (diamonds), PJH363. (C and D) *polA1,* which has the flap endonuclease, but lacks polymerase and editing functions, has no effect on adaptive amplification, and shows an approximately 3-fold increase in point mutation. Thus, the flap endonuclease promotes amplification, and the function that depresses point mutation is either polymerase or editing. Results of two independent isolates and two independent isogenic wild-type control strains are shown. Strains used are as follows: Pol^+^ (triangles, and squares with crosses), PJH356 and PJH401; and *polA1* (circles and squares)*,* PJH353 and PJH399.

Pol I consists of three main domains: an N-terminal flap-endonuclease domain (also called a 5′ exonuclease) homologous to human FEN1, a polymerase domain, and a C-terminal 3′-exonuclease editing domain. We dissected the roles of Pol I by using mutant alleles of *polA* that lack specific activities. Figure 4A shows a 5-fold reduction in amplification in the *polA107* mutant, which lacks only the flap-endonuclease activity [50], although point mutation is unchanged (Figure 4B). Previously, Nagata et al. [51] showed an effect of *polA107* on +1 but not −1 frameshift mutation, in agreement with our finding of no effect of this allele on −1 point mutations in the Lac system. In repeated experiments, the reduction in amplification in the *polA107* strain was 3.8-fold ± 0.6 (mean ± SEM for three experiments). We showed that the lower amplification was not caused by a reduced ability of *polA107* strains to form colonies in the timescale of the experiment by performing a reconstruction experiment (as above for *rec* and *ruvC* strains). For *polA107,* the mean time to colony formation for five different amplicons was 3.8 ± 0.1 d, whereas the time for Pol^+^ strains was 3.6 ± 0.06 d. We demonstrated a similar absence of growth delay during adaptive mutation experiments for a *polA12*Ts strain previously [32]. The depression in amplification in *polA107* is also not caused by loss of viability of the mutant cells under starvation. The number of viable cells on the plates on day 5 relative to day 1 of the starvation was 1.1 ± 0.3 (mean ± SEM of three experiments) with the lowest value being 0.8. Amplification is unchanged in a *polA1* mutant, which lacks all domains except the flap endonuclease [52] (Figure 4C), whereas point mutation shows an approximately 3-fold increase (Figure 4D). Thus, the flap endonuclease, which acts in processing Okazaki fragments, is the only activity of Pol I required for adaptive amplification. Also, different activities of Pol I affect point mutation and amplification, confirming that Pol I does not channel intermediates from one pathway to another [32], but acts separately and differently in each separate pathway.

**Figure 5:**
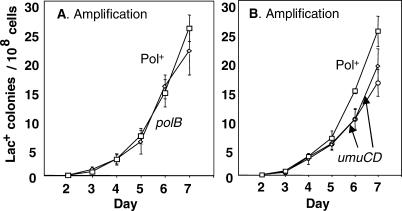
DNA Polymerases II and V Are Not Required for Adaptive Amplification (A) Deletion of *polB,* encoding Pol II, has no effect on amplification. The associated strains are as follows: Pol^+^ (squares), SMR4562; and *polB* (diamonds), SMR3661. (B) Deletion of *umuCD* has little or no effect on amplification. Results for two independent isolates are shown. Strains used are as follows: Pol^+^ (squares), SMR4562; and *umuCD* (diamonds and circles), SMR3525 and SMR3526.

Pol II and Pol V are controlled by the SOS DNA damage response, and Pol II is also expressed constitutively [53]. Pol II, encoded by *polB,* is not required for amplification (Figure 5A). (For effects of these polymerases on point mutagenesis, see [33,35,54].) Because the SOS response is not required for adaptive amplification [48], Pol V is not expected to be required. We found that Pol V, encoded by *umuCD,* does not affect total adaptive Lac^+^ colony formation [35], and when amplification was analyzed separately, we found only a very slight decrease (Figure 5B).Thus, although the lagging-strand processing activity of Pol I is required for amplification, the DNA synthesis activities of Pols I, II, IV, and V are not.

### No Involvement of Excision Repair Pathways

**Figure 6:**
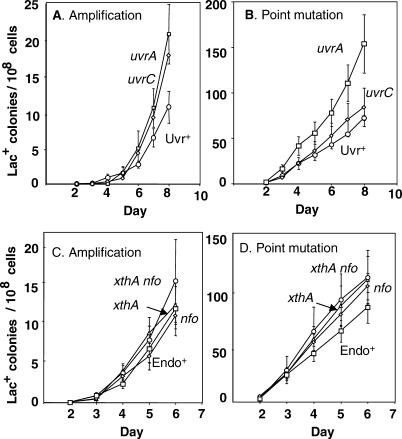
NER and BER Are Not Involved in Adaptive Amplification (A) Deletions of *uvrA* and *uvrC,* essential components of NER, do not depress adaptive amplification, or (B) point mutation. The associated strains are as follows: Uvr^+^ (circles), SMR4562; *uvrA* (squares), PJH357; and *uvrC* (diamonds), PJH358. (C) Inactivation, singly or together, of *xthA* and *nfo* genes encoding ExoIII and EndoIV, redundant AP-endonucleases required for BER, does not depress adaptive amplification, or (D) point mutation. Strains used are as follows: Endo^+^ (squares), SMR4562; *xthA* (triangles), SMR5796; *nfo* (diamonds), SMR4558; and *xthA nfo* (circles), SMR5952.

The main roles of Pol I flap endonuclease are in the removal of RNA primers and the processing of Okazaki fragments during lagging-strand synthesis, and in two DNA-repair pathways: nucleotide excision repair (NER) and base excision repair (BER). Using deletions of *uvrA* and *uvrC,* we show that NER (reviewed by [55]) is not required for amplification or point mutation (Figure 6A and 6B). An alternative enzyme to UvrC, encoded by *cho,* is unlikely to be involved because it is SOS inducible, and its activity requires UvrA [56]. Mutations in genes encoding the two major AP endonucleases ExoIII and EndoIV (reviewed by [53]) separately or together, have no effect on amplification, showing that BER is also not required for adaptive amplification (Figure 6C). This supports a role in lagging-strand processing for Pol I in adaptive amplification.

**Figure 7:**
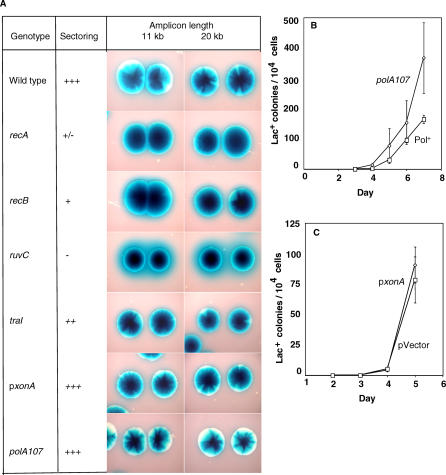
Reduced Amplification in a *polA107* Mutant and in Cells Overexpressing *xonA* Is Not Caused by an Inability to Expand from a Duplication to Give Lac^+^ Colonies (A) Colony-sectoring phenotypes of amplified DNA arrays in relevant genetic backgrounds: a qualitative measure of homologous recombination in repeated sequences. Phenotypes were scored by visual inspection of sectoring in 3-d-old colonies of these strains grown on M9 glucose minimal medium containing 60 μg/ml X-gal. Use of minimal medium is preferable to rich medium because the former avoids the presence of sectors caused by loss of the F′ (F^−^ derivatives of these strains are proline requiring). Before plating, the cells were grown in lactose minimal medium, selecting for maintenance of the amplification. Sectoring was scored on a five-point scale (+++, ++, +, +/−, and −) based on the intensity of sectoring, and the occurrence of non-sectored colonies among more than 100 colonies derived from single cells. This provides a measure of recombination that leads to loss of amplification [16]. The 11-kb and 20-kb amplifications used are from independent, representative strains PJH18 and PJH51 carrying *lac* amplifications with 10.88-kb and 20.33-kb repeat unit lengths, respectively. Strains used, containing amplicons from PJH18 and PJH51 respectively, were: Wild type; PJH428 and PJH430, *recA;* PJH433 and PJH435, *recB;* PJH438 and PJH440, *ruvC;* PJH443 and PJH445, *traI;* PJH453 and PJH455, p*xonA;* PJH518 and 519, *polA107;* PJH520 and PJH521. (B and C) Quantitative measure of expansion from a duplication to amplification. Cells of the genotypes shown carrying a 25.5-kb duplication of the *lac* region were plated under conditions of an adaptive mutation experiment. (B) A duplication-carrying *polA107* strain is at least as well able as the isogenic *polA^+^* strain to amplify and form colonies. Strains used are as follows: *polA107* (diamonds), PJH522; and Pol^+^ (squares), PJH407. (C) The presence of *xonA,* encoding ExoI, expressed from a plasmid has no effect on the ability of a duplication-carrying strain to amplify and form colonies. The associated strains are as follows: p*xonA* (diamonds), PJH523; and pVector (squares), PJH525.

### To Which Phase of the Amplification Process Do Genetic Requirements Apply?

A plausible mechanism of amplification formation involves an initial tandem duplication event, followed by expansion of the duplication to form an amplified array of many copies of the repeat unit. For the stress-induced amplifications described here, the initial duplication event must be a non-homologous recombination event, because the junctions contain only microhomologies (Table 1 and Figure 2), and we propose below that this occurs by a template-switching mechanism (Results/Discussion). Any given genetic requirement for amplification might pertain to either or both of these phases. To attribute the involvement of Pol I and ExoI to the duplication formation (non-homologous recombination) phase, we therefore need evidence that their involvement is not exclusively at the expansion (homologous recombination) phase. Some data to this effect already exist. First, as discussed below (Results/Discussion) ExoI 3′-SS exonuclease affects homologous recombination oppositely to that shown here for amplification; ExoI is a requirement that is partially redundant with 5′-SS exonucleases in homologous recombination [57,58], and not an inhibitor of the process as seen here for amplification (Figure 3). This argues that 3′-ends are most important here in formation of the initial duplication. Following the suggestion [16] that expansion and breakdown of the amplified array occur by the same mechanism, possibly by unequal crossing-over, we can obtain further evidence by studying the instability of the amplification in different genetic backgrounds.

It was reported previously that when amplified isolates are engineered to become *recA*-defective, sectoring of amplified colonies is strongly reduced [16]. This is taken to mean that RecA is required for breakdown of the array, and therefore also for expansion of the array. This does not exclude an involvement of RecA at other stages. Figure 7A shows the degree of sectoring observed for each of the mutant backgrounds that were shown here to affect amplification, for two different amplified arrays. We find that components of the RecBC double-strand break-repair system (RecA, RecBC and RuvC) are needed for sectoring. Thus we postulate that these are involved in expansion, which is supported by their known protein functions in homologous recombination. They could also act in events leading to the initial duplication (discussed below). Importantly, strains with the *polA107* mutation, which lacks Okazaki-fragment-processing flap-endonuclease activity, and strains that overproduce ExoI 3′-SS exonuclease, are positive for sectoring (Figure 7A), and so presumably also for the expansion phase of amplification. This implies that that both of these defects in amplification occur at the stage of non-homologous recombination to form an initial duplication.

As a more rigorous, quantitative measure, we show for both a *polA107* strain and for a strain over-producing ExoI*,* that their ability to expand a duplication into amplification is at least as good as that of the isogenic *polA^+^* and vector carrying control strains. We did this by providing a duplication and following the ability of various strains to form colonies on lactose minimal medium, which requires amplification of the duplication. As seen Figure 7B and 7C, the *polA107* strain expands slightly better than the Pol^+^ strain, while expansion in the strain overproducing ExoI from a plasmid is indistinguishable from that of the strain carrying the control plasmid (pVector). These results imply that both the *polA107-* and ExoI-overproduction-caused defects in amplification occur at the stage of non-homologous recombination to form an initial duplication.

### Further Discussion

The findings reported strongly constrain possible molecular mechanisms for stress-induced amplification. In aggregate, our results imply the involvement of three specific DNA intermediates—double-strand DNA ends, 3′-single-strand ends, and lagging-strands—and suggest a particular long-distance template-switch model, which we propose below. We also found that stress-induced *lac* amplifications are similar to previous specially-selected promoter-fusion amplifications [19], thought to be spontaneous. This supports the ideas both that those previous results are general, and that the amplification observed might have been stress-induced amplification, potentially formed by the same mechanism being described here. This would suggest that most genome rearrangements might occur during stress, provoking genome evolution most often when cells are poorly adapted to their environments. This property, and the mechanism, may apply to many organisms and circumstances.

### Molecular Mechanism: Previous General Models Excluded

Many (but not all, see [6]) models for the mechanism of tandem gene amplifications include an initial recombination event to generate a duplicated DNA segment, followed by unequal crossing over between sister DNA molecules to expand the repeat to high-level amplification (reviewed [6]). First, some have suggested that the initial duplications in stress-induced amplification would form by homologous recombination between repeated sequence elements present in the E. coli genome [38]. We find no evidence to support this model. The only repetitive DNA sequence that is represented among the junctions is the REP sequences, which comprise families of 38 bp imperfect intergenic palindromes [59], and these appear not to be represented preferentially or to have undergone homologous exchanges (Results/Discussion, Figure 2, Table S1).

Second, several other possible models involve use of particular DNA sequences in the initial recombination event that generates a duplication [39]. We found no similarity to known consensus sequences such as topoisomerase binding sites, or palindromes, at which the rearrangements might be initiated. There is also no other strong consensus among the junction sequences, except for their guanine-richness (38.5% versus 27.6% in this region generally) and the frequent, but not universal association with the 5′GTGG3′ and 5′CTGG3′ sequences, discussed below.

### DNA Intermediates: 3′-Single-Strand Ends Implicated

3′-SS-DNA ends, and not 5′-SS ends, are implicated as intermediates in stress-induced amplification by the findings that amplification increases in strains lacking ExoI 3′-SS-specific exonuclease, decreases when ExoI is overproduced, and is unaffected by 5′-SS exonuclease RecJ (Figure 3). The increase implies that ExoI normally prevents some amplification that would have been provoked had 3′-SS ends not been removed by ExoI. The decrease upon ExoI overproduction implies that in wild-type cells, 3′-SS ends are critical intermediates in stress-induced amplifications.

3′-SS ends could be critical either in formation of the initial duplication by “nonhomologous” recombination, or its amplification by homologous recombination, or both. Three lines of evidence support the idea that 3′-SS ends are most important in formation of the initial duplication. First, overproduction of ExoI 3′-SS exonuclease has little or no effect on visible sectoring of colonies carrying amplification (Figure 7A), which is caused by homologous recombination between the repeats. Second, overproduction of ExoI does not effect amplification of a constructed duplication (Figure 7C). These both imply that the critical step at which 3′-SS ends are intermediates is in formation of an initial duplication, not subsequent expansion by homologous recombination between repeats. Third, the initial duplication is the nonhomologous recombination event, which we will illustrate below, is most likely to occur by a template-switch mechanism. In previous rearrangements (deletions) ascribed to template-switching, which occur at microhomologies very similar to those seen here, removal of ExoI also stimulated the events [47]. These deletions were RecA-independent and had no component of homologous recombination. By contrast, RecA- RecBC-dependent homologous recombination is affected oppositely by loss of ExoI (and RecJ). Removal of neither exonuclease alone stimulated recombination, whereas removal of both simultaneously *decreased* recombination [57,58]. The similarity of our results with ExoI to those with microhomology-mediated rearrangements [47], and not with homologous recombination [57,58], suggests that the 3′-SS-end intermediates important in stress-induced amplification are critical at the stage of generation of the initial duplication. We will suggest that 3′-SS ends act in a template-switch mechanism between previously non-contiguous DNA segments at distant replication forks.

### DNA Intermediates: Lagging Strands

Lagging strands at replication forks are implicated as intermediates in stress-induced amplification by the requirement for the Pol I 5′-flap endonuclease function (Figure 4), which acts in processing of Okazaki fragments, and by the fact that Pol I-dependent NER and BER pathways are not required for amplification (Figure 6). The possibility that Pol I acts at formation of the initial duplication (non-homologous recombination) rather than homologous expansion is supported by the lack of a sectoring (unequal recombination) phenotype of the *polA107,* flap-endonuclease–defective mutant strain and by the finding that in a strain carrying a duplication at *lac,* Lac^+^ colonies form as well or better than in the Pol^+^ strain (Figure 7).

Second, also implicating lagging strands as intermediates in the mechanism of stress-induced amplification is the association of the novel junctions with the sequence 5′GTGG3′ (Table 1, Figure 2, Table S1). We find this sequence inside, overlapping, or within 5 bp of the junction sequence in 16 of the 31 sequences, and two more have it within 20 bp. Eight of the sixteen have the sequence two or three times within 20 bp. This sequence was reported previously to be associated with template-slippage mutation (a polymerase-error process distinct from template switching discussed here) which occurs in strains lacking the polymerase activity of DNA Pol I ([60] and references therein). Although the role of the sequence is not known, it is suggested to be a polymerase pause site, perhaps for the endings of Okazaki fragments [60].

**Figure 8:**
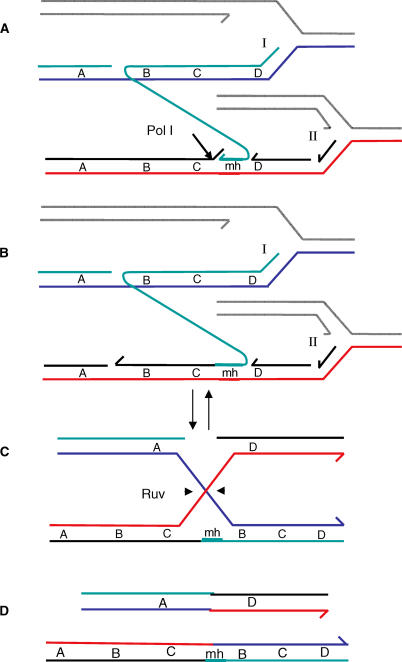
Long-Distance Template Switching**:** A Possible Molecular Mechanism for Formation of Repeats DNA replication forks are proposed to stall during the stress of starvation because of insufficient dNTPs (deoxyribonucleotide triphosphates), or uncorrected template lesions, or other conditions caused by stress-induced depletion of resources. We propose, for two reasons, that these replication forks might be initiated by DSBR. First, normal replication origins are not expected to fire during starvation, when origin-specific controls should maintain their inactivity [70]. Second, double-strand breaks induce adaptive amplification strongly [37], implying that DSBR is part of the mechanism, and promotes amplification at a rate-limiting step. (A) A nascent lagging strand from stalled replication fork I anneals to the lagging strand template of another replication fork (II) ahead of fork I, aided by the flap-endonuclease function of Pol I cutting at the arrow, forming a microhomology junction. The two forks are moving in parallel on sister molecules. (B) The microhomology junction might be stabilized by extension by a DNA polymerase (version I of this model, shown here). In version II of this model (not shown), immediately after the 5′-flap endonuclease removes the strand segment needed for the 3′ terminus to pair, the microhomology junction is stabilized by simple ligation to the 5′ end of the Okazaki fragment ahead of it. (C) The exchange is isomerized to reveal a Holliday junction that can be cleaved by RuvC Holliday-junction endonuclease (arrowheads). Resolution of the Holliday junction as shown yields the products shown in (D). (D) One of the products formed by resolution of the Holliday junction carries a duplication of the sequence “BC,” whereas the other carries a reciprocal deletion. The alternative non-crossover resolution does not yield a duplication. “A,” “B,” “C,” and “D” indicate discrete sequences on the chromosome. “mh” indicates the microhomology at the novel junction. Each line represents a single nucleotide chain or strand. Different strands are distinguished by color. Half arrows on strands indicate 3′ ends. The gray strands are those that are not involved in the rearrangement.

Third, the related sequence 5′CTGG3′ (found in or near 15 of the 31 junction sequences) has been implicated in sites of nonhomologous recombination causing deletion and duplication in the human α-gal A gene [61]. The 5′CTG3′ trinucleotide was identified as the minimal primase binding site in phage G4 [62], suggesting a relationship to the ends of Okazaki fragment as in the model proposed here (Figure 8). 5′CTG3′ occurs 54 times among the 617 possible trinucleotides in 22 of the 31 junction sequences and their flanking sequences illustrated in Table S1. Using a figure 0.552 for the GC content of this region, 11.4 occurrences of 5′CTG3′ are expected. This difference is highly significant (contingency chi-squared = 29 for one degree of freedom: *p* < 0.001). The complementary trinucleotide 5′CAG3′ is also over-represented, occurring 27 time in 17 junctions, so that 27 of 31 junctions have either 5′CTG3′ or its complement. Thus there is a strong correlation between the occurrence of this putative primase-binding trinucleotide and the junction sequences. This suggests that the nonhomologous recombination events occur preferentially near to the ends of Okazaki fragments.

Finally, if the new template following the switch is a lagging-strand template, the free 3′ that switches template must also be from a nascent lagging strand. If the nascent leading strand were to switch templates to the lagging-strand template, this would produce an inversion. All but one (Figure 2C) of the 31 amplicons studied are simple direct repeats, excluding this possibility. The isolation of one amplified array carrying an inversion shows that other configurations also occur. However, inverted template switching is unlikely to lead to a viable Lac^+^ product unless a second inversion occurs, as apparently happened in the one case that we saw. We conclude that the majority of the proposed template switch events would involve a switch of a nascent lagging strand to a lagging strand template.

### DNA Intermediates: Double-Strand Ends

DNA double-strand ends (DSEs) and their repair by homologous recombination are implicated in stress-induced amplification by its requirements for the DSE-specific DSBR protein RecB, and the other DSBR proteins, RecA, and RuvC (Figure 1). We show elsewhere that DSEs, induced in vivo by the restriction enzyme I-*Sce*I, stimulate stress-induced amplification [37]. Frequent DSEs in this assay system are likely to originate from SS-nicks made at the transfer origin of the F′ by TraI endonuclease, which is required for stress-induced amplification (Figure 1). These SS-nicks could become DSEs via replication or other mechanisms (reviewed [37]). The requirement for TraI in stress-induced amplification (and point mutation) can be substituted for by an I-*Sce*I cut, further supporting this idea [37].

### Template-Switching Events

The gist of template switching models for “nonhomologous” recombination and genome rearrangement is that a 3′-SS primer terminus leaves its correct spot at a replication fork and alights in a different (template) region of the replication fork by means of base-pairing with limited complementary base sequences. The switch produces a novel junction and the rearrangement (deletion, duplication, inversion, etc.).

The novel junctions associated with stress-induced amplification are predominantly guanine-rich simple direct microhomology repeats (Table 1, Figure 2 **)**. Such junctions have been reported in several other instances of nonhomologous recombination in *E. coli,* including spontaneous amplification [19], duplication [20], deletion [63], and rearrangement [64]. They share many features and can be considered together as representing the same range of mechanisms [39]. These are considered most likely to involve template-switching [39] based on the following features [39]: (i) The recombination events are independent of RecA, and (ii) are altered by conditional mutations in DNA replication genes. (iii) Mutations affecting such rearrangement processes have not easily been found by transposon-insertion mutagenesis, implying essential functions, such as replication genes. (iv) Some of these rearrangements were affected by changes in 3′-SS exonucleases [47], and (v) methyl-directed mismatch repair [65]; and (vi) all previously (except amplification) have shown a very strong distance dependence; they occurred between sequences hundreds to a few thousand bp apart, and were essentially never seen at distances over 10 kb (references in [39]). The reason for this distance dependence is presumably that a template switch is most likely to occur within a single replication fork [39].

Our data are compatible with several aspects of previously-described rearrangements that suggest a template-switching event in the stress-induced amplification mechanism, except for the distance dependence. The average length of amplicons appearing from day 3 to day 9 can be estimated from published data to be 20 kb, with a modal value estimated to be 16 kb [24], so that there is no suggestion that amplicons of less than 10 kb are preferred. This suggests a different kind of template-switch mechanism, which we suggest (below) may be specific to stress-induced chromosome instability.

### Molecular Mechanism: A Long-Distance Template-Switch Model

We suggest the following model for stress-induced amplification in *E. coli:* double-strand breaks, resulting mainly from the action of TraI, are repaired by DSBR via formation of repair replication forks. We suggest that these forks are likely to stall because the cells are starved for carbon, and that fork stalling is favored because they are repair-replication forks. That is, whereas controls on standard origins should disfavor initiation under starvation/stationary-phase conditions, repair forks must operate, and so could be more prone to stalling for want of a sufficient supply of dNTPs or because of unrepaired lesions in the templates. This induces the rearrangement specifically under stress. Fork stalling results in the production of free 3′-DNA ends by dissociation of nascent DNA ends. A possible sequence of events for how these ends might lead to duplication then expansion is depicted in Figure 8.

In Figure 8, a free 3′-DNA end from the lagging strand might alight on a template strand present in a nearby fork, thus switching template (see Figure 8A). In this model, the switch occurs between different sequence regions in two replication forks running in parallel on sister molecules (Figure 8A). The microhomology is sufficient to allow the 3′ end to form a stable joint only if the Pol I 5′-flap endonuclease is present (see Figure 8A) to remove the 5′-flap of the next Okazaki fragment in the fork onto which the primer has switched, so that this 5′ end does not compete with the switching 3′ end for binding to the template (Figure 8A). In version I of this model (Figure 8B), the switched strand might need to be extended after the Pol I flap endonuclease removes the competing 5′ end of the next Okazaki fragment, in order to stabilize the nonhomologous joint. In version II of this model, not shown, extension synthesis might not be necessary; simple ligation of the primer-3′ end to the 5′ end of the Okazaki fragment in front of it will also work. This could explain the lack of requirement for the Pol I synthesis domain (Figure 4C), which normally works with the 5′-flap endonuclease function in nick translation of Okazaki fragments (nick translation is 5′-flap endonuclease coupled to 3′-extension synthesis), and the lack of need for Pols IV [48], II, and V (Figure 5). Versions I and II both lead to Figure 8C in which the switched-strand intermediate can be isomerized to reveal a Holliday junction. Endonucleolytic resolution of that junction by the Ruv system (Figure 8C) can produce products, one of which carries the duplication, which can be of any size (Figure 8D), depending on where the primer terminus joins the second fork.

The model illustrated in Figure 8 achieves duplication of a length of the genome essentially by capture of a segment of DNA from a sister molecule. Other possible models could achieve duplication by re-replication of a length of DNA: for example, by replicating a segment that was already synthesized, in the switching strand, after it switches to the new template. If one adheres to the premise (above) that both the switching strand and the new template molecule are lagging strands/lagging-strand templates, then models of the re-replication type are more complex than the mechanism illustrated.

The unique property of this model that allows relative distance independence of recombination between the microhomologies is that the switch occurs between templates at two replication forks at different positions, instead of at one fork (in previous rearrangements/models [39]). This, and the need for subsequent homologous recombination to complete the amplification event, distinguishes this from the mechanisms of previously described microhomology-mediated rearrangements in *E. coli,* which are distance dependent and so probably occur within a single replication fork, and occur independently of homologous recombination proteins.

### Predictions for Other Rearrangements

The scale of the rearrangements found in human cancers precludes events confined to a single replication fork; so it is likely that, if template-switching underlies such changes in mammalian cells, switching between forks is the norm. Recent characterization of double-minute chromosomes in human gliomas showed all amplicons to be in the megabase range, and six of seven analyzed showed microhomology of 2, 3, or 5 bp at the novel junctions [14], suggesting a similarity to the mechanism studied here. Similarly, some copy number variations in the human genome show junction sequences similar to those seen here, coupled with repeat lengths too long to be caused by template switching within a single replication fork (see [30]). These, as well as the many ubiquitous copy number variations not yet examined for junction sequences (see [3–5]), could form similarly to the bacterial amplifications studied here.

In contrast with the requirement for the flap endonuclease for stress-induced amplification (Figure 4), deletion of the flap-endonuclease domain of *polA* [66], or analogous flap endonuclease functions from yeast *(rad27)* [67], or human *(fen1)* [68], are associated with *increased* microsatellite (oligonucleotide repeat) instability. Microsatellite instability is associated with accelerated tumor progression in mice heterozygous for a *fen1* mutation [69]. So, apparently, loss of the flap endonuclease promotes short-range slippage reactions, but its presence appears to be required for long-distance template-switch interactions.

### Stress Inducibility

An important aspect of the amplifications studied here is their demonstrated stress inducibility. They occur after prolonged starvation [24], and require some function(s) induced in the RpoS general-, stationary-phase- and starvation-stress response [25]. These amplifications may represent an important mechanism for inducible adaptation to environmental stress, potentially accelerating evolution specifically when cells are poorly adapted to their environments or under stress.

Our data make plausible the possibility that the majority of amplifications in E. coli are stress induced. *lac* amplifications selected as specific promoter fusions also show very similar microhomology junctions and sizes of amplicons [19]. Our data indicate, first, that those previous junction structures were a general feature of E. coli amplification, not specific to their selection. Second, the data support the possibility that perhaps these amplifications were also induced by stress of selection, forming only after cells experience the stress of starvation on lactose medium, as those authors suggested.

The specific feature of the mechanism proposed that causes the amplifications to be formed preferentially under stress is the fork-to-fork long-distance template switch, promoted by fork stalling. We suggest that “unscheduled” (origin-independent) repair replication might be particularly prone to fork stalling during starvation and stress because of lack of controls exerted on replication origins that prevent replication initiation during starvation/stress [70].

Stress-induced amplification, and the stress-induced aspects of it in particular, may provide important models for genomic instability in general [24]. Enhanced rearrangement under stress, via mechanisms similar to that discussed here, might underlie genome instabilities such as seen in cancers [1,2] and experienced in tumor microenvironments [71]. The enzymatic DNA repair activities are very well conserved [72], and similar mechanisms may probably underlie observed amplification and chromosomal instability events common in many cancers. Such mechanisms may pertain also to the origins of rearrangements that create the copy number variations, both pathogenic and polymorphic, frequently observed in human genomes [3–5,29] .

## Materials and Methods

All strains used are derivatives of FC40 [33] or the isogenic strain SMR4562 [35]. Except as described below, mutations were moved into FC40 or SMR4562 from other strains by standard phage P1–mediated transduction. Where a linked transposon was used to move a mutant allele, the isogenic “wild-type” control strains used carry the same transposon. The strains used, and their origins, are listed in Table 2. Genotypes were confirmed by phenotypic tests and, where necessary, by sequencing.

The novel junctions of amplified arrays were localized by PCR with a series of outward-facing primers. The location of the junction was further refined by restriction analysis of the PCR product, and finally identified by sequencing. Sequencing was performed by Lone Star Labs (Houston, Texas, United States).

A duplication of the *lac* region consisting of 25,429 bp from *prpC* to *mhpT* including a *cat* gene from pACYC184 was made by the technique of Slechta et al. [73]. To determine the ability of strains to expand the duplication into an amplified array, very pale colonies from rich medium with chloramphenicol and X-gal were grown for 2 d in minimal medium with glycerol and chloramphenicol, then plated to lactose minimal medium as in an adaptive mutation experiment, and colonies were counted daily.

Media and experimental procedures were as described [32]. In the experiments reported, the number of Lac^−^ viable cells did not change by a factor of two or more during the relevant time period. All amplification experiments have been repeated with comparable results. Error bars on the graphs of amplification rates are SEM of three or four cultures.

## Supporting Information

Table S1 Novel Junction Sequences in Their Contexts (40 KB DOC)

## Abbreviations

BER: base excision repair
bp: base pair
DSBR: double-strand break repair
DSE: double-strand end
kb: kilobase
NER: nucleotide excision repair
SEM: standard error of the mean
SS: single strand
