# Supplementary material for: On the Mechanism of Gene Amplification Induced under Stress in Escherichia coli
Source: PLoS Genet. 2006 Apr 7;2(4):e48. doi: 10.1371/journal.pgen.0020048 (PMC1428787; doi:10.1371/journal.pgen.0020048)
Supplement: Supplementary file 1 [file pgen.0020048.st001.doc]

**Supplementary Table 1. Novel junction sequences in their contexts**

PJH1	13.69 kb.

____________*mhpA*

tca**A**ag**CC**ca**GTGGCTGG**tagcctgt**G**a

tca**A**ag**CC**ca**GTGGCTGG**ctgttaac**G**g

gtt**A**tt**CC**gt**GTGGCTGG**ctgttaac**G**g

*codB____________*

PJH3	18.36 kb.

____________*mhpE*

t**T**at**G**tg**G**tg**GATTCTGGCGGT**gcga**T**gaaca t**T**at**G**tg**G**tg**GATTCTGGCGGT**cgcc**T**tgggg a**T**tgGgt**G**gc**GATTCTGGCGGT**cgcc**T**tgggg

*prpR_____________*

PJH4	20.31 kb.

____________*mhpT*

attgcggc**G**g**GTGGC**atc**G**cccagg

attgcggc**G**g**GTGGC**gat**G**tttgcc

tggcgtcg**G**t**GTGGC**gat**G**tttgcc

*prpR____________*

PJH13	11.68 kb.

____________*mhpF*

**CG**g**T**ctgg**C**g**CGCGCCA**gacgt**A**tggg

**CG**g**T**ctgg**C**g**CGCGCCA**ataac**A**gaat

**CG**a**T**acta**C**c**CGCGCCA**ataac**A**gaat

*lacA____________*

PJH18	10.88 kb.

____________*mhpA*

a**A**a**TCCA**gca**GTGGCGCG**cgttgg**GC**ac

a**A**a**TCCA**gca**GTGGCGCG**atgacc**GC**ca

c**A**t**TCCA**act**GTGGCGCG**atgacc**GC**ca

*cynT_____________*

PJH19	22.58 kb.

____________*mhpE*

ggtttgcaag**GTTCCAGCT**tt**A**a**CC**ta**T**g

ggtttgcaag**GTTCCAGCT**cg**A**c**CC**cg**T**c

ccggaactca**GTTCCAGCT**cg**A**c**CC**cg**T**c

*prpE_____________*

PJH22 and PJH31	11.27 kb.

____________*mhpC*

caaact**GG**tg**CTGATGGGCGGCGGT**ac**G**ggc**GG**ca caaact**GG**tg**CTGATGGGCGGCGGT**gg**G**ctt**GG**tg gtccgc**GG**ct**CTGATGGGCGGCGGT**gg**G**ctt**GG**tg

*cynX_____________*

PJH23 and PJH37	23.36 kb.

____________*mhpE*

ttg**A**a**G**cg**G**c**GGCG**g**ATG**tg**GTG**a

ttg**A**a**G**cg**G**c**GGCG**a**ATG**aa**GTG**t

aac**A**c**G**gc**G**g**GGCG**a**ATG**aa**GTG**t

*prpC___________*

PJH26	9.50 kb.

____________*mhpA*

tcc**TC**gaa**A**t**GCGCTTC**aa**GCCGA**t**GC**

tcc**TC**gaa**A**t**GCGCTTC**ct**GCCGA**c**GC**

ggg**TC**agc**A**g**GCGCTTC**ct**GCCGA**c**GC**

*cynS____________*

PJH28	20.78 kb.

____________*mhpE*

c**A**c**G**c**C**a**A**aa**TCGCG**ac**GTTG**ttg**C**

c**A**c**G**c**C**a**A**aa**TCGCG**tc**GTTG**cgc**C**

g**A**t**G**t**C**g**A**tt**TCGCG**tc**GTTG**cgc**C**

*prpC____________*

PJH29	19.95 kb.

____________*mhpE*

**AA**aacag**C**gg**TCTGGCTGGA**agt**C**gaag**G**c

**AA**aacag**C**gg**TCTGGCTGGA**gaa**C**cgct**G**g

**AA**gtgct**C**ta**TCTGGCTGGA**gaa**C**cgct**G**g

*prpE____________*

PJH30	16.61 kb.

____________*mhpR*

gcgtact**G**ct**GGCGGGCGATGCC**g**C**gca**C**at**C**a

gcgtact**G**ct**GGCGGGCGATGCC**a**C**ttc**C**cg**C**g

aatcctg**G**gc**GGCGGGCGATGCC**a**C**ttc**C**cg**C**g

*prpE____________*

PJH32	20.27 kb.

____________________________REP between *mhpE* and *mhpT*

**GC**g**CA**cgactgccggat**G**cg**GC**gtgaa**CG**c**CTTATC**c**GGCCTAC**gg**A**a**TG**gcg**T** **GC**g**CA**cgactgccggat**G**cg**GC**gtgaa**CG**c**CTTATC**c**GGCCTAC**cc**A**c**TG**ttt**T** **GC**t**CA**gatgcctgatgc**G**ac**GC**tgacg**CG**t**CTTATC**a**GGCCTAC**cc**A**c**TG**ttt**T**

REP between *prpE* and *codB________*

PJH39	15.85 kb.

____________*mhpA*

**G**ct**T**tgttgc**CGCGCCACCAC**ttccg**C**aaac **G**ct**T**tgttgc**CGCCACCGCCA**cctgc**C**cttt **G**aa**T**gacaaa**CGCCACCGCCA**cctgc**C**cttt

*prpE* (inverted)____

**and**	7.85 kb.

*prpE* (inverted)_*___*

atg**CG**gt**G**gc**GCTGGCG**ac**C**tcga**T**g**G**

cgt**CG**tg**G**cg**GCTGGCG**ac**C**tcga**T**g**G**

cgt**CG**tg**G**cg**gctggcg**tt**C**cttg**T**c**G**

*____________lacA*

PJH49	8.98 kb.

____________*mhpR*

attgacgtgc**TGGTGG**tggagaaac**T**

attgacgtgc**TGGTGG**aatgtcttt**T**

ccacgacgtt**TGGTGG**aatgtcttt**T**

*lacA__________*

PJH51	20.33 kb.

____________*mhpF*

gac**T**t**C**cagt**GCGCTGGCGAC**ag**CG**gaaa**A**a gac**T**t**C**cagt**GCGCTGGCGAC**ct**CG**atgg**A**c ata**T**g**C**ggtg**GCGCTGGCGAC**ct**CG**atgg**A**c

*prpE_____________*

PJH59 and PJH65	15.53 kb.

_________________REP between yaiL and *yaiM*

ggcg**A**agt**GT**a**G**ac**CGGATAAGGCG**t**TC**a**CGCCGCATCCG**g**CA**g**T**cg**T**gc ggcg**A**agt**GT**a**G**ac**CGGATAAGGCG**c**TC**g**CGCCGCATCCG**a**CA**t**T**ga**T**tg tatg**A**tat**GT**t**G**gt**CGGATAAGGCG**c**TC**g**CGCCGCATCCG**a**CA**t**T**ga**T**tg

REP between *lacA* and *lacY* ____________________

PJH62	15.89 kb.

____________*mhpA*

**T**g**A**tcgcgta**CTGCTGGCGGG**cgat**GC**cg**C**g **T**g**A**tcgcgta**CTGCTGGCGGG**gatg**GC**ga**C**t **T**t**A**cgcgccg**CTGCTGGCGGG**gatg**GC**ga**C**t

*prpE____________*

PJH63	12.25 kb.

____________*mhpR*

a**G**t**GG**tgc**G**a**TGCAGGCC**g**CTG**ag**T**t**CC**

a**G**t**GG**tgc**G**a**TGCAGGCC**a**CTG**gt**T**a**CC**

t**G**g**GG**att**G**c**TGCAGGCC**a**CTG**gt**T**a**CC**

*codB____________*

PJH64	8.89 kb.

____________*mhpA*

gg**C**ta**T**aaca**GTGGTAT**g**C**g**C**ga**C**g**C**c

gg**C**ta**T**aaca**GTGGTAT**t**C**a**C**tc**C**c**C**g

tc**C**gg**T**tcgc**GTGGTAT**t**C**a**C**tc**C**c**C**g

*cynX_____________*

PJH69	13.36 kb.

____________*mhpE*

tat**TT**atgtg**GTGG**a**TT**ctg**G**cg**GTGC**gat**G**aa

tat**TT**at---**GTGG**t**TT**tct**G**gc**GTGC**tgc**G**ta ggc**TT**gcccc**GTGG**t**TT**tct**G**gc**GTGC**tgc**G**ta

*cynX_______________________*

PJH74	32.28 kb.

____________*mhpF*

**AAA**tg**G**ct**G**a**GGCGGTGCAG**ag**C**g**G**a**A**aaa

**AAA**tg**G**ct**G**a**GGCGGTGCAG**gc**C**c**G**c**A**cgg

**AAA**at**G**gc**G**t**GGCGGTGCAG**gc**C**c**G**c**A**cgg

*yahJ_____________*

PJH75	19.06 kb.

____________*mhpT*

ggca**GG**ggtg**ATGTTTGCC**ctgca**A**at**GG**

ggca**GG**ggtg**ATGTTTGCC**agttg**A**tg**GG**

atgg**GG**ctgc**ATGTTTGCC**agttg**A**tg**GG**

*codA_____________*

PJH76	21.16 kb.

____________*mhpA*

cgaag**C**gaag**CACTCTCC**g**G**tcggcaa**G**

cgaag**C**gaag**CACTCTCC**a**G**gtaaagc**G**

tatgt**C**tcta**CACTCTCC**a**G**gtaaagc**G**

*prpB____________*

PJH77	26.24 kb.

____________*mhpE*

**CA**g**C**ct**CGC**g**GGAATGG**gcg**C**gg**G**cgc

**CA**g**C**ct**CGC**g**GGAATGG**ttt**C**ct**G**ata

**CA**c**C**ag**CGC**t**GGAATGG**ttt**C**ct**G**ata

*prpR____________*

PJH78	22.98 kb.

____________*mhpE*

**AC**tatggttt**CGGCGCACAT**agcgaccttg

**AC**tatggttt**CGGCGCACAT**tatcgaacaa

**AC**cggctggg**CGGCGCACAT**tatcgaacaa

*prpE_____________*

PJH83

__________*cynT*	9.48 kb

aa**A**aa**GT**gt**-GCGCGG**gc**T**ttg**C**t**G**t**G**tcgc

aa**Aa**a**GT**gtc**GCGCGG**ct**T**aac**C**c**G**c**G**gtct

tt**A**tt**GT**aa-**GCGCGG**ct**T**aac**C**c**G**c**G**gtct

*mhpR___________________*

PJH165	13.89 kb.

REP between *mhpE* and *mhpT____*

gcgg**GAA**t**TTGTAGGCCTGATAAG**acgc**G**ttagc gcgg**GAA**t**TTGTAGGCCTGATAAG**cgca**G**cgtat cgct**GAA**c**TTGTAGGCCTGATAAG**cgca**G**cgtat

between *cynX* and *lacA____________*

The junctions are listed under the strain number of the strain that carries them. Amplicon lengths in kb are based on the published sequence of *E. coli* wild-type strain MG1655^1^. For each junction, the upper and lower sequences show the sequences that interacted. These sequences are distinguished by color, red or blue. The middle sequence is the novel sequence. The regions in which these sequences occur are given by the gene designation above and below, except the REP sequences, which are intergenic. REP sequences are underlined. Microhomology regions at the novel junctions common to both sequences are in black and all base identities are in upper-case letters. Relative deletions are indicated by dashes.

1.	Blattner, F. R. et al. The complete genome sequence o*f Escherichia co*li K-12*. Scien*c**e 2**77, 1453-1474 (1997).
